# Inclusion of hnRNP L Alternative Exon 7 Is Associated with Good Prognosis and Inhibited by Oncogene SRSF3 in Head and Neck Squamous Cell Carcinoma

**DOI:** 10.1155/2019/9612425

**Published:** 2019-11-13

**Authors:** Lingfeng Xu, Jiaoxiang Shen, Jun Jia, Rong Jia

**Affiliations:** ^1^The State Key Laboratory Breeding Base of Basic Science of Stomatology (Hubei-MOST) & Key Laboratory of Oral Biomedicine Ministry of Education, School & Hospital of Stomatology, Wuhan University, Wuhan, China; ^2^Department of Orthodontics, Xiamen Stomatology Hospital, Hospital and School of Stomatology, Xiamen Medical University, Xiamen, China; ^3^Department of Oral and Maxillofacial Surgery, School and Hospital of Stomatology, Wuhan University, Wuhan 430079, China

## Abstract

**Background and Objectives:**

Alternative splicing is increasingly associated with cancers. HnRNP L is a splicing factor that promotes carcinogenesis in head and neck squamous cell carcinoma (HNSCC) and other cancers. Alternative exon 7 of hnRNP L contains an in-frame stop codon. Exon 7-included transcripts can be degraded via nonsense-mediated decay or encode a truncated hnRNP L protein. Exon 7-excluded transcripts can encode full-length functional hnRNP L protein. HnRNP L has an autoregulation mechanism by promoting the inclusion of its own exon 7. This study aimed to understand the relationship between the alternative splicing of exon 7 and HNSCC. Oncogenic splicing factor SRSF3 has an alternative exon 4 and similar autoregulation mechanism. HnRNP L promotes SRSF3 exon 4 inclusion and then inhibits SRSF3 autoregulation.

**Materials and Methods:**

The relationship between alternative splicing of hnRNP L exon 7 and clinical characteristics of HNSCC in a TCGA dataset was analyzed and confirmed by RT-PCR in a cohort of 61 oral squamous cell carcinoma (OSCC) patients. The regulators of exon 7 splicing were screened in 29 splicing factors and confirmed by overexpression or silencing assay in HEK 293, CAL 27, and SCC-9 cell lines.

**Results:**

The inclusion of hnRNP L exon 7 was significantly negatively associated with the progression and prognosis of HNSCC, which was confirmed in the cohort of 61 OSCC patients. SRSF3 inhibited exon 7 inclusion and hnRNP L autoregulation and then promoted the expression of full-length functional hnRNP L protein. SRSF3 exon 4 inclusion was correlated with hnRNP L exon 7 inclusion in both HNSCC and breast cancer. HNSCC patients with both low hnRNP L exon 7 and SRSF3 exon 4 inclusion show poor overall survival.

**Conclusions:**

Inclusion of hnRNP L alternative exon 7 is associated with good prognosis and inhibited by oncogene SRSF3 in HNSCC.

## 1. Introduction

Alternative splicing of pre-mRNA allows one gene to express multiple protein isoforms with different functions [1]. However, when misregulated, cancer cells use this mechanism to produce proteins with deleted, added, or altered functional domains, resulting in tumorigenesis. Dysregulated alternative splicing of pre-mRNA is tightly associated with cancers [2–5]. A complete understanding of this misregulation may reveal some valid drug targets for therapeutic intervention.

**Figure 1:**
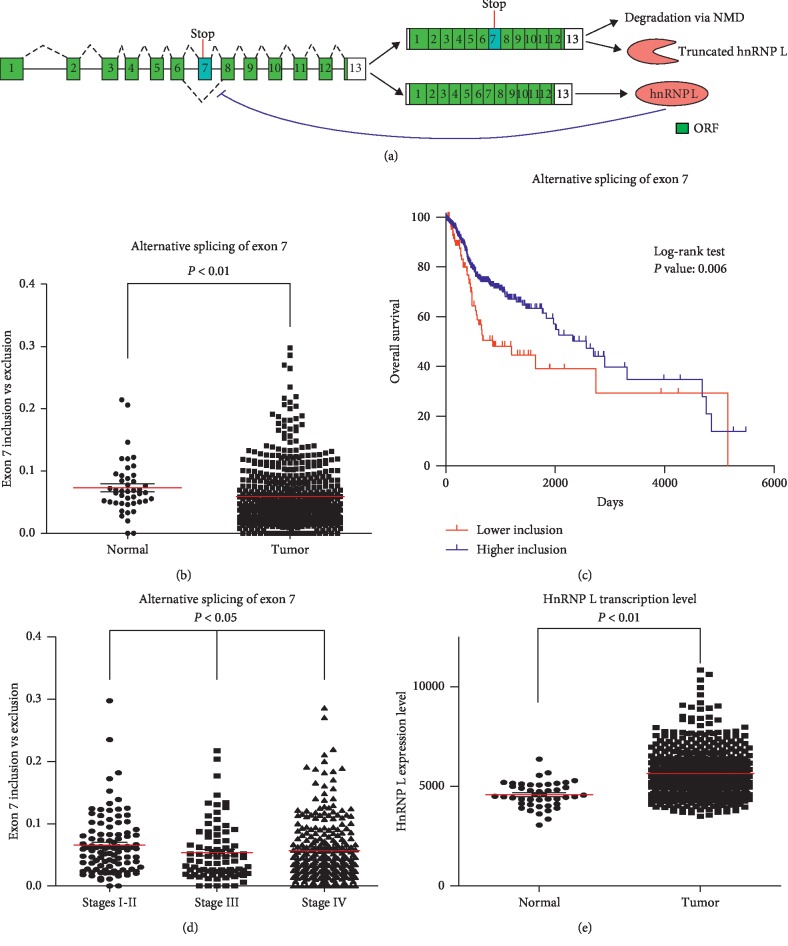
The inclusion of hnRNP L exon 7 in head and neck cancer is significantly changed. (a) Schematic diagram of the alternative splicing of hnRNP L exon 7. Human hnRNP L pre-mRNA has an alternative exon 7, which contains an in-frame stop codon. Exon 7-included transcripts are degraded by NMD or encode truncated hnRNP L protein. Exon 7-excluded transcripts can encode full-length hnRNP L. hnRNP L can promote the inclusion of exon 7 and autoregulate its expression. Boxes and lines represent exons or introns in the pre-mRNA, respectively. Dashed lines above or below introns indicate RNA splicing direction. (b-e) The alternative splicing of hnRNP L exon 7 in the TCGA head and neck cancer patients. Normalized expression levels of exon 7-excluded or exon 7-included isoforms were obtained from an online program, TSVdb. (b) The ratios of hnRNP L exon 7 inclusion versus exclusion isoform were compared between normal (44 cases) and tumor (520 cases) tissues. (c) Kaplan-Meier curves of overall survival for patients with low ratio (84 cases) or high ratio (356 cases) of exon 7 inclusion versus exclusion isoform. Low ratio was defined as less than mean-0.8 SD (standard deviation). (d) The ratios of hnRNP L exon 7 exclusion versus inclusion isoform in stages I-II (101 cases), stage III (81 cases), or stage IV (265 cases). (e) Normalized total hnRNP L transcriptional levels were compared between normal and tumor tissues.

Recent studies have shown that several splicing factors are involved in the tumorigenesis of head and neck squamous cell carcinoma (HNSCC) (including oral squamous cell carcinoma (OSCC), a subtype of HNSCC) [5–9]. Two groups of splicing factors play important roles in alternative splicing of pre-mRNA, heterogeneous nuclear ribonucleoproteins (hnRNPs), and serine-arginine-rich (SR) proteins. HnRNP L is present in the nucleoplasm as part of the heterogeneous nuclear ribonucleoprotein (HNRNP) complex, which is involved in nearly every step in mRNA expression and biogenesis, including IRES-mediated translation [10, 11], splicing regulation [12, 13], transport of intronless mRNAs [14, 15], and mRNA stability [16, 17]. Previous studies have shown that hnRNP L promotes the proliferation, invasion and metastasis of OSCC [7], non-small-cell lung cancer [18], and breast cancer [19] cells. Knockdown of hnRNP L significantly inhibits hepatocellular carcinoma cell migration, growth, and invasion in vitro [1]. Knockout of hnRNP L alters hematopoiesis and is lethal in mice [20]. HnRNP L has two transcript variants, which are generated by alternative splicing of exon 7. Exon 7 has an in-frame stop codon. Therefore, the longer isoform with exon 7 is degraded via nonsense-mediated decay (NMD) ([Supplementary-material supplementary-material-1]) or encodes a truncated hnRNP L protein (almost undetectable by Western blot). By contrast, the shorter isoform without exon 7 can encode full-length functional hnRNP L protein (Figure 1(a)). HnRNP L has an autoregulation mechanism by promoting its own exon 7 inclusion to maintain a relatively stable expression level of hnRNP L in cells ([Supplementary-material supplementary-material-1]) [13]. Exon 7 is the key to control the expression of full-length oncogenic hnRNP L. In our previous study, we demonstrated that hnRNP L protein is overexpressed in OSCC tissues and cells [7]. However, the relationship between the alternative splicing of hnRNP L exon 7 and OSCC remains unclear.

SRSF3 (previously known as SRp20) is the smallest member of the SR protein family and plays important roles in regulating alternative RNA splicing [4, 21, 22]. SRSF3 is a proto-oncogene that is upregulated in various types of cancer, including OSCC [23–26]. Overexpression of SRSF3 induces varied cancerous phenotypes, such as cell cycle progression, antiapoptosis, and cell proliferation. Our previous research has indicated that the alternative splicing of SRSF3 exon 4 is regulated by hnRNP L [7]. In the present study, we found that the inclusion of hnRNP L exon 7 in HNSCC is significantly lower than that in normal tissues and is positively correlated with patients' good prognosis. Moreover, the inclusion of hnRNP L exon 7 is negatively regulated by SRSF3. SRSF3 and hnRNP L can mutually inhibit their autoregulation and promote the expression of both their functional and oncogenic isoforms. Alternative splicing of hnRNP L exon 7 may be a new therapeutic target of HNSCC or OSCC treatment.

## 2. Materials and Methods

### 2.1. TCGA Data Analysis

We analyzed the TCGA dataset of head and neck squamous cell carcinoma (44 normal and 520 primary cancer cases). The expression of hnRNP L and SRSF3 in patients was measured by mRNA sequencing. Normalized expression levels of isoforms were obtained from TSVdb (an online program, http://www.tsvdb.com) to calculate the ratios of alternative exon inclusion isoform versus exclusion isoform. A low or high level of the ratio of alternative exon inclusion versus exclusion was determined by the number of standard deviations (SD) from the mean. To analyze the correlation between the ratios of alternative exon inclusion versus exclusion and the survival of patients, we downloaded the overall survival data from TSVdb and analyzed them with Graphpad Prism.

### 2.2. Cells and Plasmids

HEK 293 and CAL 27 cells were maintained in our lab. HEK 293 and CAL 27 cells were cultured in Dulbecco's modified Eagle medium (DMEM) containing 10% fetal bovine serum (FBS, HyClone) and 1% antibiotic-antimycotic (Invitrogen, USA). SCC-9 cells were cultured in a 1 : 1 mixture of DMEM and Ham's F12 medium containing 10% FBS, 400 ng/mL hydrocortisone, and 1% antibiotic-antimycotic. For cycloheximide (CHX) treatment, cells were treated with CHX (100 *μ*g/mL, Sigma, USA) for 5 hours. DMSO was used as control. T7-tagged hnRNP L (T7-hnRNP L) overexpression plasmid was constructed in our previous study [7]. The T7-tagged SRSF3 (T7-SRSF3) expression plasmid was nicely provided by Dr. Zheng Zhi-Ming. Plasmid transfection was performed in the presence of Lipofectamine 2000 (Invitrogen, USA) in accordance with the manufacturer's instructions.

### 2.3. siRNA and Transfection

The sequences of SRSF3 siRNAs are 5′AGAGCUAGAUGGAAGAACA3′ (si#1) and 5′GGAAAUAGAAGACAGUUUG3′ (si#2). The sequence of hnRNP L siRNA is 5′CUACGAUGACCCGCACAAA3′. The siRNAs were synthesized in GenePharma (China). Cells were transfected with 20 nM siRNA in the presence of Lipofectamine 2000 in accordance with the manufacturer's protocol. Nonspecific siRNA (NC) was used as control. Cells were transfected with siRNAs twice in an interval of 48 h. At 96 h after the first transfection, total proteins or RNAs were collected by 2× sodium dodecyl sulfate sample buffer or the total RNA miniprep kit (Axygen, USA).

### 2.4. Western Blot

Total protein samples were separated by 10% SDS-PAGE gel and transferred to a PVDF membrane. The following antibodies were used on Western blot: mouse anti-SRSF3 (clone 3G271, Santa Cruz Biotechnology, USA), mouse monoclonal anti-hnRNP L (clone 4D11, Santa Cruz Biotechnology), or horseradish peroxidase-labeled mouse anti-*β*-actin antibody (Sigma-Aldrich, USA).

### 2.5. RT-PCR

The cDNA was reverse-transcribed from the total RNA using Maxima H Minus Reverse Transcriptase (Thermo Fisher Scientific, USA). PCR amplification was performed with 2 x Taq Master Mix (Vazyme, NJ, USA) using the listed primers: 5′-ACGCCATCTTTCAGAACTGTGCT-3′ and 5′-CATATTCTGCGGGGTGATCTC-3′ for hnRNP L exon 7 inclusion detection, 5′-ATCAGGATACTTGGGACTACACAAAC-3′ and 5′-CATCATGGTAATGGCTGTGGTAC-3′ for hnRNP L exon 7 exclusion and inclusion detection, 5′-GGAGTCCTCCACCTCGTCGCA-3′ and 5′-ACGAGACCTAGAGAAGGATCGGGAC-3′ for SRSF3 exon 4 splicing detection, and 5′-GAAGGTGAAGGTCGGAGTC-3′ and 5′-GAAGATGGTGATGGGATTTC-3′ for GAPDH.

### 2.6. Patients and Tissue Samples

Sixty-one patients diagnosed with OSCC in School and Hospital of Stomatology in Wuhan University were involved in this study. All histologic diagnoses were performed by the pathology department in School and Hospital of Stomatology, Wuhan University. The clinicopathological characteristics of patients are listed in [Supplementary-material supplementary-material-1]. Informed consent was obtained from all participants. The experimental protocol was approved by the Ethics Committee at the School and Hospital of Stomatology in Wuhan University.

### 2.7. Statistical Analysis

The ratios of hnRNP L exon 7 inclusion versus exclusion isoform were compared between groups using the nonparametric Mann–Whitney *U* test in SPSS software. All two-group statistical comparisons of means were performed with Student's *t*-test (GraphPad Prism). Survival analysis was performed with a log-rank test. Correlation between the alternative splicing of exons was analyzed with Spearman rank correlation. The quantification of RT-PCR was calculated using Quantity One software. Statistically significant value was considered at *p* < 0.05.

## 3. Results

### 3.1. Alternative Splicing of hnRNP L Exon 7 Is Significantly Associated with the Clinic Characteristics of Head and Neck Cancer

We analyzed the alternative splicing of exon 7 in head and neck cancer or normal tissues in the TCGA database from the online program TSVdb. The inclusion of exon 7 was significantly lower in cancer tissues than in normal tissues (Figure 1(b)). Moreover, patients with low inclusion of exon 7 showed significantly poor overall survival (Figure 1(c)). Patients with clinical pathological stage I or II showed significantly higher inclusion of exon 7 compared with those with stage III or IV (Figure 1(d)). These results suggest that the inclusion of hnRNP L exon 7 is negatively associated with the initiation, progression, and prognosis of head and neck cancer. In addition, in line with our previous publication [7], we found that the total hnRNP L mRNA levels were significantly higher in head and neck cancer tissues than in normal tissues (Figure 1(e)). In consideration that the inclusion of exon 7 causes the degradation of hnRNP L mRNA, the decreased inclusion of exon 7 contributes to the increased level of hnRNP L mRNA in cancer tissues.

**Figure 2:**
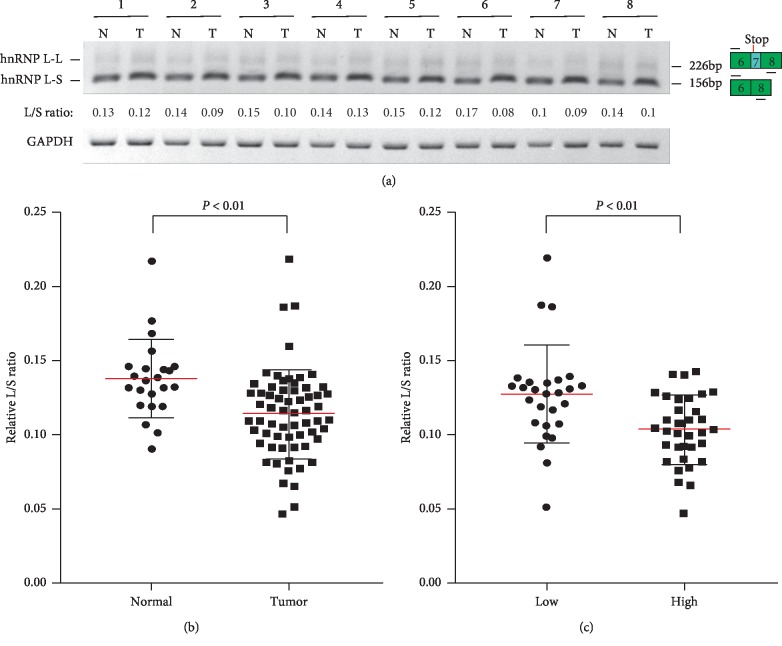
The lower inclusion of hnRNP L exon 7 was confirmed in 61 OSCC cancer samples compared with 23 adjacent normal tissues. (a) The representative RT-PCR results of the alternative splicing of hnRNP L exon 7 in normal or OSCC tissues. HnRNP L-L and hnRNP L-S represent transcripts with or without exon 7, respectively. Diagrams on the right of show the structures of hnRNP L spliced products and primer positions (short lines above or below exons). (b) The scatter dot plot summarized the relative inclusion levels of hnRNP L exon 7 (L/S ratio) in OSCC tumor and normal tissues. (c) The scatter dot plot summarized the relative inclusion levels of hnRNP L exon 7 (L/S ratio) in OSCC tumors with low (28 cases) or high (33 cases) pathological stage.

To confirm the results in the TCGA database, we analyzed the alternative splicing of hnRNP L exon 7 in 61 OSCC patients or 23 normal controls by RT-PCR. As expected, the inclusion of exon 7 is also significantly lower in OSCC patients than normal controls (Figures 2(a) and 2(b) and [Supplementary-material supplementary-material-1]). In addition, patients with low pathological stage showed significantly higher inclusion of exon 7 than those with high pathological stage (Figure 2(c)).

### 3.2. Alternative Splicing of hnRNP L Exon 7 Is Regulated by SRSF3

**Figure 3:**
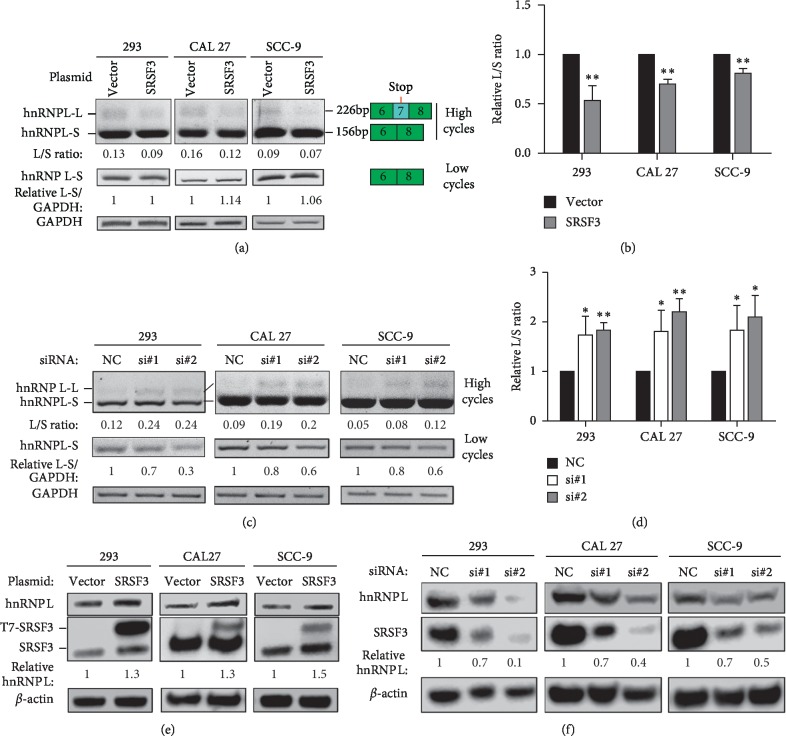
SRSF3 inhibits the inclusion of hnRNP L exon 7. (a) HEK 293, CAL 27, or SCC-9 cells were transfected by SRSF3 expression or vector control plasmid. The alternative splicing of exon 7 was analyzed by RT-PCR. The L/S ratio represents the ratio of exon 7 inclusion versus exclusion isoform. (b) The histogram summarized the effects of SRSF3 overexpression on the alternative splicing of hnRNP L exon 7 in HEK 293, CAL 27, and SCC-9 cells. Data are the means ± SD, *n* = 3. (c) Knockdown of SRSF3 promotes the inclusion of hnRNP L exon 7 in HEK 293, CAL 27, and SCC-9 cells. Cells were transfected with anti-SRSF3 siRNA (si#1 or si#2) or nonspecific control siRNA (NC). The alternative splicing of exon 7 was analyzed by RT-PCR. ^*∗*^: *P* < 0.05, ^*∗∗*^: *P* < 0.01. (d) The histogram summarized the effects of SRSF3 knockdown on the alternative splicing of hnRNP L exon 7 in HEK 293, CAL 27, and SCC-9 cells. Data are the means ± SD, *n* = 3. (e-f) The overexpression of T7-tagged SRSF3 (e) or knockdown efficiency of SRSF3 (f) was confirmed by Western blot. The expression of hnRNP L full-length protein was also analyzed by Western blot. “Relative hnRNP L” represents the relative expression level of hnRNP L normalized by *β*-actin and controls.

We then determined how the alternative splicing of hnRNP L exon 7 is regulated. We overexpressed 29 splicing factors in 293 cells ([Supplementary-material supplementary-material-1]) and analyzed the alternative splicing of exon 7 by RT-PCR. SRSF3 showed a greater decreased ratio of exon 7 inclusion versus exclusion ([Supplementary-material supplementary-material-1]) compared with the control. We also overexpressed SRSF3 in OSCC cell lines, CAL 27 and SCC-9, and confirmed that the upregulation of SRSF3 can significantly decrease the inclusion of exon 7 (Figures 3(a) and 3(b) and [Supplementary-material supplementary-material-1]). Furthermore, we silenced SRSF3 expression in these three cell lines with siRNAs. As expected, downregulation of SRSF3 significantly increased the inclusion of exon 7 (Figures 3(c) and 3(d) and [Supplementary-material supplementary-material-1]). The overexpression or silencing of SRSF3 in these cell lines was confirmed by Western blot (Figures 3(e) and 3(f)). In line with RT-PCR result, the overexpression or silence of SRSF3 also increased or decreased the protein levels of hnRNP L full-length function protein (Figures 3(e) and 3(f)). The expression of truncated hnRNP L encoded by transcripts containing exon 7 was below the detectable level. These results suggested that SRSF3 promotes the exclusion of exon 7 and inhibits hnRNP L autoregulation.

**Figure 4:**
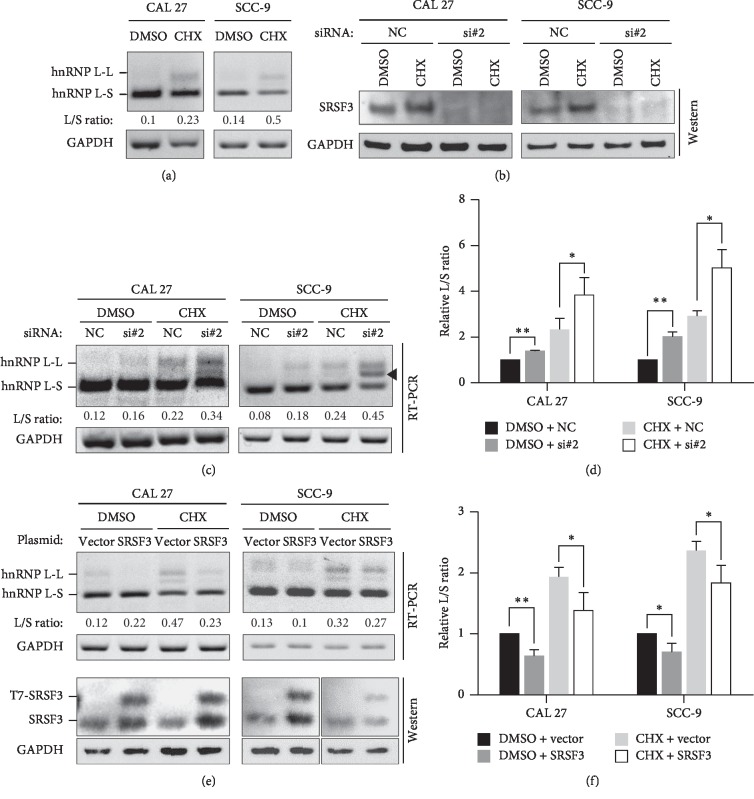
The inhibition of SRSF3 on the inclusion of hnRNP L exon 7 was evaluated by CHX treatment. (a) CHX was used to block NMD. Cells were treated with CHX or DMSO for 5 hours. The alternative splicing of exon 7 was analyzed by RT-PCR. L/S ratio represents the ratio of exon 7 inclusion versus exclusion isoform. (b-c) CAL 27 or SCC-9 cells were treated with anti-SRSF3 siRNA (si#2) or nonspecific control siRNA (NC), followed by CHX treatment. Knockdown efficiency of SRSF3 was analyzed by Western blot (b). Alternative splicing of hnRNP L exon 7 was analyzed by RT-PCR (c). ◄: hybridization product of both long and short isoforms. (d) The histogram summarized the effects of SRSF3 knockdown on the alternative splicing of hnRNP L exon 7 in CAL 27 and SCC-9 cells. Data are the means ± SD, *n* = 3. ^*∗*^: *P* < 0.05, ^*∗∗*^: *P* < 0.01. (e) CAL 27 or SCC-9 cells were transfected with SRSF3 expression plasmid or vector control plasmid, followed by CHX treatment. The alternative splicing of exon 7 was analyzed by RT-PCR. Overexpression of SRSF3 was confirmed by Western blot. (f) The histogram summarized the effects of SRSF3 overexpression on the alternative splicing of hnRNP L exon 7 in CAL 27 and SCC-9 cells. Data are the means ± SD, *n* = 3.

Exon 7-included isoform is the target of NMD. To precisely evaluate the inhibition of SRSF3 on the inclusion of hnRNP L exon 7, cells were treated with CHX for NMD inhibition. CHX treatment significantly increased the levels of exon 7-included isoform compared with DMSO control treatment (Figures 4(a) and [Supplementary-material supplementary-material-1]). Moreover, after CHX treatment, SRSF3 knockdown also showed significantly upregulated levels of exon 7-included isoform in both CAL 27 and SCC-9 cells (Figures 4(b)–4(d)). Overexpression of SRSF3 also showed significantly decreased levels of exon 7-included isoform in both CAL 27 and SCC-9 cells (Figures 4(e) and 4(f)). These results confirmed that SRSF3 promotes the exclusion of hnRNP L exon 7.

### 3.3. SRSF3 and hnRNP L Mutually Inhibit Their Autoregulation

**Figure 5:**
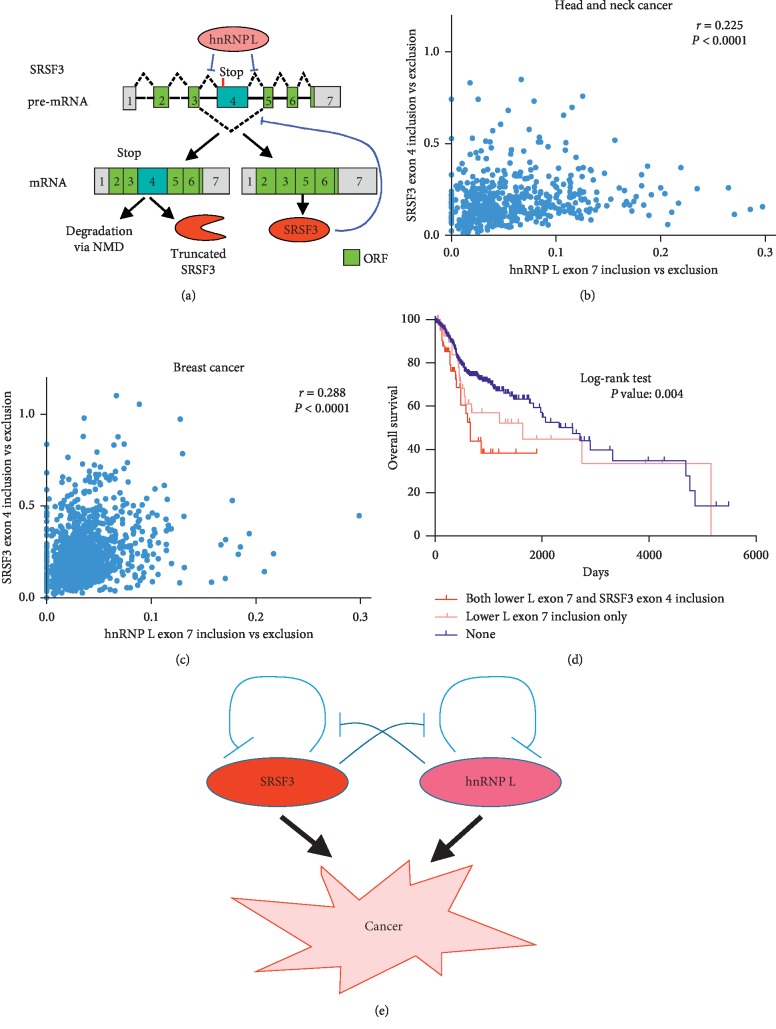
(a) The model of autoregulation of SRSF3 expression is regulated by hnRNP L. SRSF3 has an alternative exon 4, which has an in-frame stop codon. The longer isoform with exon 4 is degraded via NMD or encodes a truncated SRSF3 protein. The shorter isoform without exon 4 can encode full-length functional SRSF3 protein. SRSF3 promotes the inclusion of its own exon 4 to maintain relatively stable levels of SRSF3 in cells. HnRNP L inhibits the inclusion of exon 4. (b-c) The correlation between the ratios of SRSF3 exon 4 inclusion versus exclusion and hnRNP L exon 7 inclusion versus exclusion in the TCGA head and neck cancer dataset (520 cases) and breast cancer dataset (1093 cases). (d) Kaplan-Meier curves of overall survival for patients with both low hnRNP L exon 7 and SRSF3 exon 4 inclusion (41 cases), low hnRNP L exon 7 inclusion only (43 cases), or both not low hnRNP L exon 7 and SRSF3 exon 4 inclusion (none, 297 cases). Low inclusion of hnRNP L exon 7 was defined as less than mean-0.8SD (standard deviation). Low inclusion of SRSF3 exon 4 was defined as less than mean-0.66SD. (e) The model of SRSF3 and hnRNP L mutually inhibiting their autoregulation and promoting carcinogenesis.

Similar to hnRNP L, SRSF3 has an alternative exon 4, which also has an in-frame stop codon. The longer isoform with exon 4 can be degraded via NMD or encode a truncated SRSF3 protein (almost undetectable by Western blot). By contrast, the shorter isoform without exon 4 can encode full-length functional SRSF3 protein (Figure 5(a)). SRSF3 also has an autoregulation mechanism by promoting the inclusion of its own exon 4 to maintain relatively stable levels of SRSF3 in cells. In our previous study, we found that hnRNP L promotes the exclusion of SRSF3 exon 4, then inhibits autoregulation, and increases the expression of full-length functional SRSF3 [7]. These results were further confirmed in [Supplementary-material supplementary-material-1]. We speculated that the alternative splicing of SRSF3 exon 4 may be correlated to alternative splicing of hnRNP L exon 7 to allow the overexpression of both SRSF3 and hnRNP L in cancer cells. Then, we analyzed the relationship between SRSF3 exon 4 splicing and hnRNP L exon 7 splicing in the head and neck cancer data of the TCGA database. Indeed, the ratio of SRSF3 exon 4 inclusion versus exclusion is positively and significantly correlated with the ratio of hnRNP L exon 7 inclusion versus exclusion in patients with head and neck cancer (Figure 5(b)). It is also true in patients with breast cancer of the TCGA database (Figure 5(c)). In addition, we found that patients with both low hnRNP L exon 7 and SRSF3 exon 4 inclusion showed poorer overall survival than those with low hnRNP L exon 7 inclusion only or both not low hnRNP L exon 7 and SRSF3 exon 4 inclusion. The median survival times of patients with both low hnRNP L exon 7 and SRSF3 exon 4 inclusion, low hnRNP L exon 7 inclusion only, or both not low hnRNP L exon 7 and SRSF3 exon 4 are 654, 1641, or 2570 days, respectively (Figure 5(d)). This result suggested that inclusion of hnRNP L exon 7 and SRSF3 exon 4 could be useful combined biomarkers in predicting the outcomes of head and neck patients.

Combined with our previous findings, we proposed that SRSF3 and hnRNP L can mutually inhibit their autoregulation and promote the expression of both their functional full-length protein and carcinogenesis (Figure 5(e)).

## 4. Discussion

Fei et al. showed that knockdown of hnRNP L suppresses prostate cancer cell growth but exerts no effect on normal-like prostate cell growth [27]. Previously, we found that the tumorigenic capacity of OSCC cells is greatly attenuated by reducing the expression level of hnRNP L [7]. In non-small-cell lung cancer, downregulation of hnRNP L induces a complete loss of tumorigenic capacity [18]. Given the important roles of hnRNP L in cell proliferation and transformation, cells apply an autoregulation mechanism to maintain the relatively stable expression of hnRNP L via alternative splicing of its own exon 7 [13]. It seems a common mechanism in splicing factors. For example, SRSF5 has alternative 3′ splice sites in exon 6, and SRSF5 promotes the usage of the proximal 3′ splice site which results in the inclusion of an in-frame stop codon and causes RNA degradation via NMD or encodes a truncated SRSF5 protein [28]. SRSF1, SRSF2, and other SR protein members almost all have autoregulation [29]. SRSF3 autoregulates its own expression by promoting the inclusion of its alternative exon 4 [30, 31]. Previously, we found that hnRNP L inhibits SRSF3 autoregulation and promotes full-length functional SRSF3 protein expression [7]. In the present study, we further discovered that SRSF3 inhibits hnRNP L autoregulation and promotes full-length functional hnRNP L protein expression. Our data showed a novel cross-regulation between SR and hnRNP families, two major splicing factor families. Moreover, we found that patients with both lower hnRNP L exon 7 and SRSF3 exon 4 inclusion showed poorer overall survival and are a subset with poorer prognosis than all other patients.

As a splicing factor, hnRNP L regulates a number of cancer-associated alternative splicing events. For example, Goehe et al. showed that hnRNP L is required for the tumorigenic capacity of non-small-cell lung cancer cell by promoting the skipping of caspase 9 exons 3–6 and the expression of caspase 9b, an antiapoptotic isoform of caspase 9 [18]. Using RNA immunoprecipitation and next-generation sequencing assay, Fei et al. identified that a cryptic exon 2b of AR and alternative exon 6 of MYH10 are regulated by hnRNP L in prostate cancer [27]. HnRNP L also regulates the alternative splicing of its own exon 7 [13]. In the present study, we further discovered that alternative splicing of hnRNP L exon 7 is also a cancer-associated alternative splicing event. Cancer tissues have significantly less exon 7 inclusion than normal controls in both TCGA database and our cohort. Patients with lower levels of exon 7 inclusion have poor prognosis and progressed disease. In consideration that the inclusion of exon 7 blocks the expression of full-length hnRNP L protein, our results pointed out that cancer cells prefer to exclude exon 7 and express a high level of hnRNP L protein.

To restrict the expression of oncogenic full-length SRSF3 protein, we designed an antisense oligonucleotide to block an exonic splicing suppressor in SRSF3 exon 4 and significantly enhance the inclusion of exon 4 and decrease the expression of oncogenic full-length SRSF3 protein [32]. The growth of HNSCC cells was significantly inhibited by the antisense oligonucleotide. We may also search key negative regulatory motifs in hnRNP L exon 7 and design antisense oligonucleotides to promote the inclusion of exon 7, reduce the expression of full-length functional hnRNP L protein, and inhibit the growth of HNSCC cells.

So far, the function of the truncated hnRNP L protein encoded by exon 7-included isoform is unknown. Compared with full-length hnRNP L protein, truncated hnRNP L lacks the RRM3 and RRM4 domains ([Supplementary-material supplementary-material-1]). These two domains are critical for RNA binding and facilitate RNA looping of target pre-mRNA [33], and they also important for antiapoptosis function of hnRNP L [34]. Therefore, truncated hnRNP L may partially lose its oncogenic function. In addition, the mRNA expression level of exon 7-included isoform is much weaker than exon 7-excluded isoform due to the degradation through NMD (Figures 3(a) and 4(a)). We could not find truncated hnRNP L protein (around 30 kDa) by using a mouse monoclonal anti-hnRNP L antibody (clone 4D11) in Western blot either. This antibody may not recognize truncated hnRNP L protein. Further studies are required to detect truncated hnRNP L protein and analyze its function.

Previously, Jia et al. found that SRSF3 could inhibit splicing by binding to an exonic splicing suppressor [35]. By using SpliceAid online program, we found an SRSF3 binding motif, “CAUC,” in hnRNP L exon 7 ([Supplementary-material supplementary-material-1]). SRSF3 may suppress exon 7 inclusion through this motif. HnRNP L can bind “CA” rich motif and suppress exon inclusion [36]. In SRSF3 exon 4, we found two “CA” rich motifs, including “CACAACAC” and “CACACA” ([Supplementary-material supplementary-material-1]). HnRNP L may bind to these motifs and suppress exon 4 inclusion. However, further experimental evidences are required for understanding the mechanisms of SRSF3- or hnRNP L-mediated suppression of exon inclusion.

## 5. Conclusion

Inclusion of hnRNP L alternative exon 7 is associated with good prognosis and inhibited by oncogene SRSF3 in HNSCC.

## Data Availability

The data used to support the findings of this study are included within the article.

## Supplementary Materials

Supplementary Materials Figure S1: hnRNP L autoregulates its own expression by increasing the inclusion of exon 7. Figure S2: the RT-PCR results of the alternative splicing of hnRNP L exon 7 in normal or OSCC tissues. Figure S3: searching splicing factors for regulating the alternative splicing of hnRNP L exon 7. Figure S4: SRSF3 inhibits the inclusion of hnRNP L exon 7. Figure S5: hnRNP L inhibits the inclusion of SRSF4 exon 4. Figure S6: the potential binding motif of hnRNP L and SRSF3. Figure S7: alignment of full-length and truncated hnRNP L protein sequences. Table S1: clinicopathological characteristics of patient samples and L/S ratio in OSCC.

## References

[1] Yau W.-Y., Shih H.-C., Tsai M.-H., Sheu J.-C., Chen C.-H., Chow L.-P. (2013). Autoantibody recognition of an N-terminal epitope of hnRNP L marks the risk for developing HBV-related hepatocellular carcinoma. *Journal of Proteomics*.

[2] Faustino N. A., Cooper T. A. (2003). Pre-mRNA splicing and human disease. *Genes & Development*.

[3] Radhakrishnan A., Nanjappa V., Raja R. (2016). Dysregulation of splicing proteins in head and neck squamous cell carcinoma. *Cancer Biology & Therapy*.

[4] Venables J. P. (2004). Aberrant and alternative splicing in cancer. *Cancer Research*.

[5] Zhang J., Manley J. L. (2013). Misregulation of pre-mRNA alternative splicing in cancer. *Cancer Discovery*.

[6] Ishii H., Saitoh M., Sakamoto K. (2014). Epithelial splicing regulatory proteins 1 (ESRP1) and 2 (ESRP2) suppress cancer cell motility via different mechanisms. *Journal of Biological Chemistry*.

[7] Jia R., Zhang S., Liu M. (2016). HnRNP L is important for the expression of oncogene SRSF3 and oncogenic potential of oral squamous cell carcinoma cells. *Scientific Reports*.

[8] Peiqi L., Zhaozhong G., Yaotian Y., Jun J., Jihua G., Rong J. (2016). Expression of SRSF3 is correlated with carcinogenesis and progression of oral squamous cell carcinoma. *International Journal of Medical Sciences*.

[9] Shih S.-C., Claffey K. P. (1999). Regulation of human vascular endothelial growth factor mRNA stability in hypoxia by heterogeneous nuclear ribonucleoprotein L. *Journal of Biological Chemistry*.

[10] Hwang B., Lim J. H., Hahm B., Jang S. K., Lee S.-W. (2009). hnRNP L is required for the translation mediated by HCV IRES. *Biochemical and Biophysical Research Communications*.

[11] Moy J. D., Moskovitz J. M., Ferris R. L. (2017). Biological mechanisms of immune escape and implications for immunotherapy in head and neck squamous cell carcinoma. *European Journal of Cancer*.

[12] Heiner M., Hui J., Schreiner S., Hung L.-H., Bindereif A. (2010). HnRNP L-mediated regulation of mammalian alternative splicing by interference with splice site recognition. *RNA Biology*.

[13] Rossbach O., Hung L.-H., Schreiner S. (2009). Auto- and cross-regulation of the hnRNP L proteins by alternative splicing. *Molecular and Cellular Biology*.

[14] Guang S., Felthauser A. M., Mertz J. E. (2005). Binding of hnRNP L to the pre-mRNA processing enhancer of the herpes simplex virus thymidine kinase gene enhances both polyadenylation and nucleocytoplasmic export of intronless mRNAs. *Molecular and Cellular Biology*.

[15] Majumder M., Yaman I., Gaccioli F. (2009). The hnRNA-binding proteins hnRNP L and PTB are required for efficient translation of the cat-1 arginine/lysine transporter mRNA during amino acid starvation. *Molecular and Cellular Biology*.

[16] Hui J. (2003). Novel functional role of CA repeats and hnRNP L in RNA stability. *RNA*.

[17] Siegel R. L., Miller K. D., Jemal A. (2017). Cancer statistics, 2017. *CA: A Cancer Journal for Clinicians*.

[18] Goehe R. W., Shultz J. C., Murudkar C. (2010). hnRNP L regulates the tumorigenic capacity of lung cancer xenografts in mice via caspase-9 pre-mRNA processing. *Journal of Clinical Investigation*.

[19] D’Agostino L., Caracciolo V., Giordano A. (2014). NSP 5a3a’s link to nuclear-cyto proteins B23 and hnRNP-L between normal and aberrant breast cell lines. *Cell Cycle*.

[20] Gaudreau M., Grapton D., Helness A. (2016). Heterogeneous nuclear ribonucleoprotein L is required for the survival and functional integrity of murine hematopoietic stem cells. *Scientific Reports*.

[21] Ajiro M., Jia R., Yang Y., Zhu J., Zheng Z.-M. (2016). A genome landscape of SRSF3-regulated splicing events and gene expression in human osteosarcoma U2OS cells. *Nucleic Acids Research*.

[22] Wang E. T., Sandberg R., Luo S. (2008). Alternative isoform regulation in human tissue transcriptomes. *Nature*.

[23] Jia R., Li C., McCoy J. P., Deng C.-X., Zheng Z.-M. (2010). SRp20 is a proto-oncogene critical for cell proliferation and tumor induction and maintenance. *International Journal of Biological Sciences*.

[24] Lin J.-C., Lee Y.-C., Tan T.-H. (2018). RBM4-SRSF3-MAP4K4 splicing cascade modulates the metastatic signature of colorectal cancer cell. *Biochimica et Biophysica Acta (BBA)—Molecular Cell Research*.

[25] Liu X., Mertz J. E. (1995). HnRNP L binds a cis-acting RNA sequence element that enables intron-dependent gene expression. *Genes & Development*.

[26] Kuranaga Y., Sugito N., Shinohara H. (2018). SRSF3, a splicer of the PKM gene, regulates cell growth and maintenance of cancer-specific energy metabolism in colon cancer cells. *International Journal of Molecular Sciences*.

[27] Fei T., Chen Y., Xiao T. (2017). Genome-wide CRISPR screen identifies HNRNPL as a prostate cancer dependency regulating RNA splicing. *Proceedings of the National Academy of Sciences of the United States of America*.

[28] Yang S., Jia R., Bian Z. (2018). SRSF5 functions as a novel oncogenic splicing factor and is upregulated by oncogene SRSF3 in oral squamous cell carcinoma. *Biochimica et Biophysica Acta (BBA)—Molecular Cell Research*.

[29] Lareau L. F., Inada M., Green R. E., Wengrod J. C., Brenner S. E. (2007). Unproductive splicing of SR genes associated with highly conserved and ultraconserved DNA elements. *Nature*.

[30] Guo J., Jia J., Jia R. (2015). PTBP1 and PTBP2 impaired autoregulation of SRSF3 in cancer cells. *Scientific Reports*.

[31] Jumaa H., Nielsen P. J. (1997). The splicing factor SRp20 modifies splicing of its own mRNA and ASF/SF2 antagonizes this regulation. *The EMBO Journal*.

[32] Guo J., Che X., Wang X., Jia R. (2018). Inhibition of the expression of oncogene SRSF3 by blocking an exonic splicing suppressor with antisense oligonucleotides. *RSC Advances*.

[33] Zhang W., Zeng F., Liu Y. (2013). Crystal structures and RNA-binding properties of the RNA recognition motifs of heterogeneous nuclear ribonucleoprotein L. *Journal of Biological Chemistry*.

[34] Hu W., Lei L., Xie X. (2019). Heterogeneous nuclear ribonucleoprotein L facilitates recruitment of 53BP1 and BRCA1 at the DNA break sites induced by oxaliplatin in colorectal cancer. *Cell Death & Disease*.

[35] Jia R., Liu X., Tao M. (2009). Control of the papillomavirus early-to-late switch by differentially expressed SRp20. *Journal of Virology*.

[36] Rothrock C. R., House A. E., Lynch K. W. (2005). HnRNP L represses exon splicing via a regulated exonic splicing silencer. *The EMBO Journal*.

