# Supplementary material for: Inclusion of hnRNP L Alternative Exon 7 Is Associated with Good Prognosis and Inhibited by Oncogene SRSF3 in Head and Neck Squamous Cell Carcinoma
Source: Biomed Res Int. 2019 Nov 13;2019:9612425. doi: 10.1155/2019/9612425 (PMC6885243; doi:10.1155/2019/9612425)
Supplement: Supplementary Materials — Figure S1: hnRNP L autoregulates its own expression by increasing the inclusion of exon 7. Figure S2: the RT-PCR results of the alternative splicing of hnRNP L exon 7 in normal or OSCC tissues. Figure S3: searching splicing factors for regulating the alternative splicing of hnRNP L exon 7. Figure S4: SRSF3 inhibits the inclusion of hnRNP L exon 7. Figure S5: hnRNP L inhibits the inclusion of SRSF4 exon 4. Figure S6: the potential binding motif of hnRNP L and SRSF3. Figure S7: alignment of full-length and truncated hnRNP L protein sequences. Table S1: clinicopathological characteristics of patient samples and L/S ratio in OSCC. [file 9612425.f1.zip › 9612425.f1/supplementary figures.pdf]

Figure S1

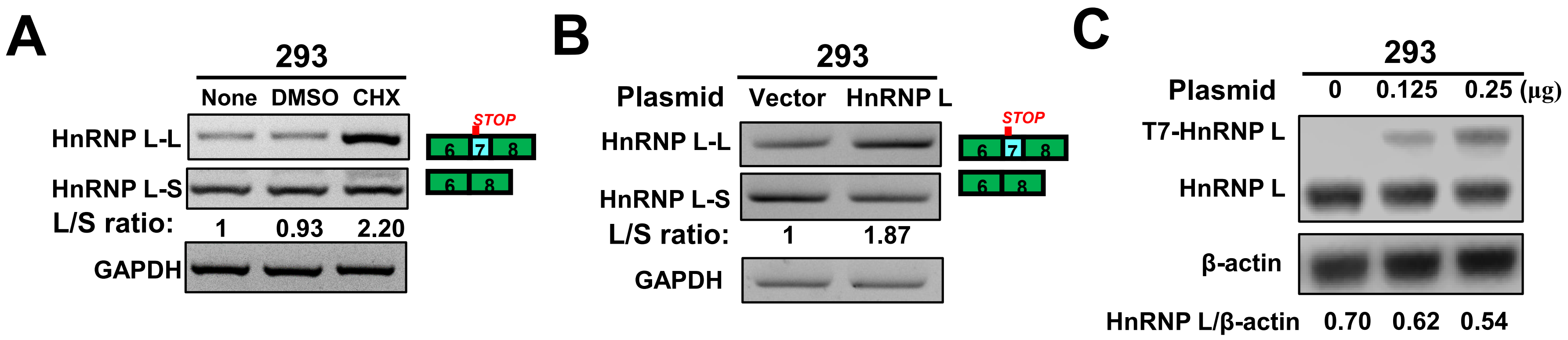

Figure S1. HnRNPL autoregulates its own expression by increasing the inclusion of exon 7. (**A**) Transcripts with exon 7 arethe targets of nonsense mediated decay (NMD). Cycloheximide (CHX) was used to block NMD. HEK 293 cells were treated with CHX or DMSO for 6 hours. The inclusion of HnRNPL exon 7 was analyzed by RT-PCR. (**B**) Overexpression of hnRNPL increased the inclusion of exon 7. (**C**) HEK 293 cells were transfected with 0, 0.125 μg, or 0.25 μg T7-tagged hnRNPL expression plasmid. The expression of endogenous hnRNPL protein was analyzed by western blot.

Figure S2

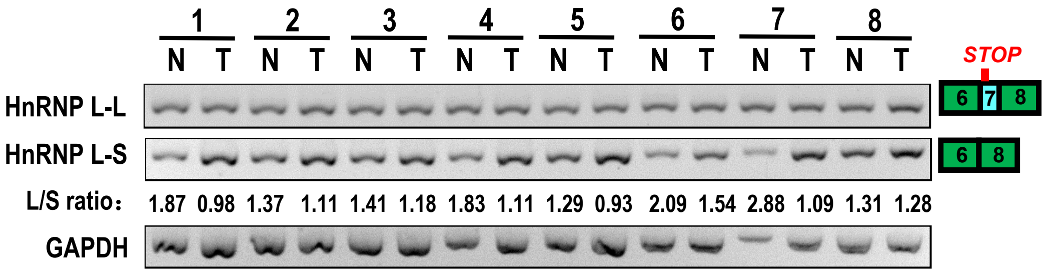

Figure S2. The RT-PCR results of the alternative splicing of hnRNP L exon 7 in normal or OSCC tissues by using two pairs of primers for isoforms with exon 7 (HnRNP L-L) or without exon 7 (hnRNP L-S), respectively.

Figure S3

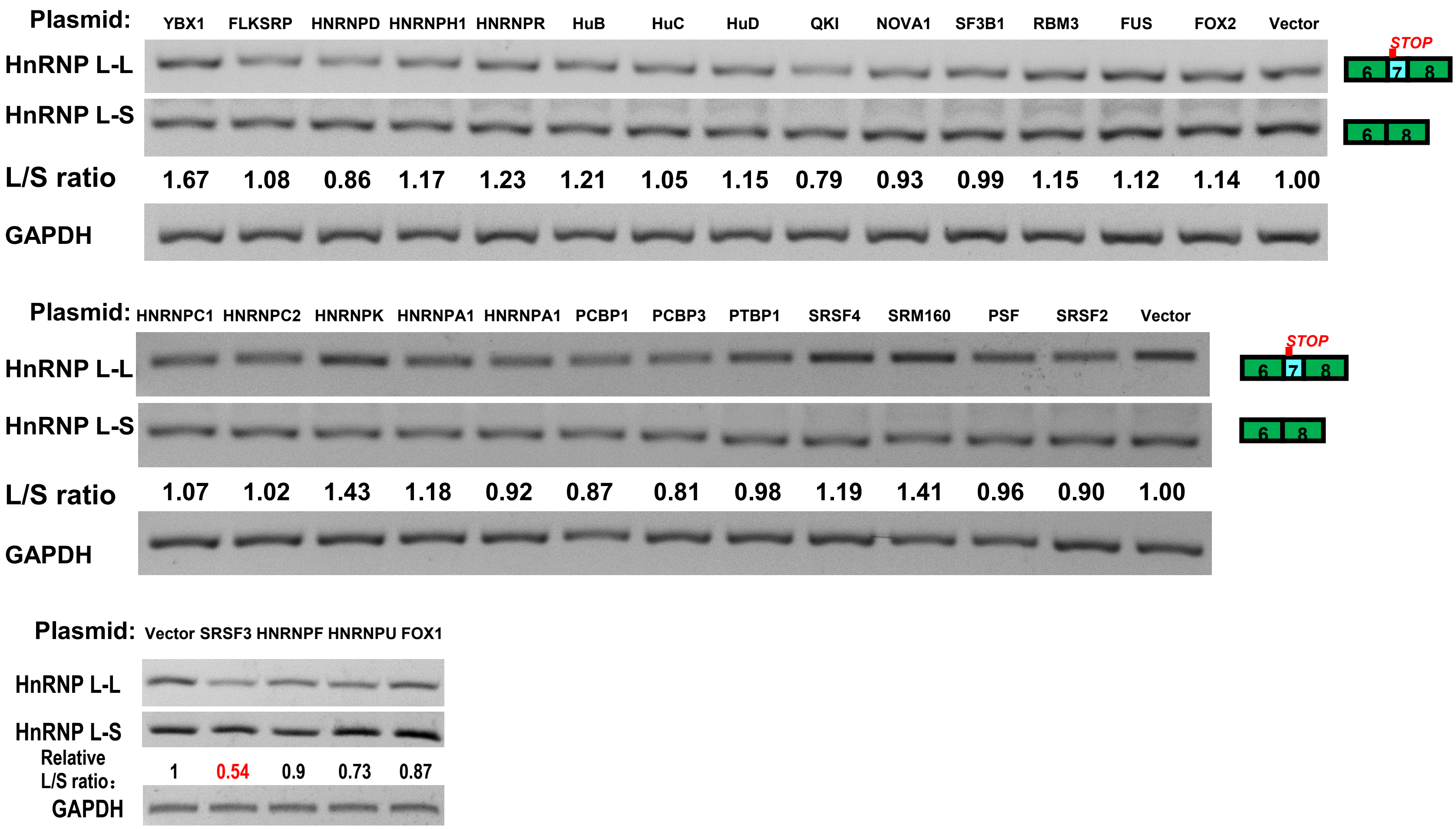

Figure S3. Searching splicing factors for regulating the alternative splicing of hnRNP L exon 7. HEK 293 cells were transfected with splicing factor expression plasmids or control vector. The inclusion of hnRNP L exon 7 was analyzed by RT-PCR.

Figure S4

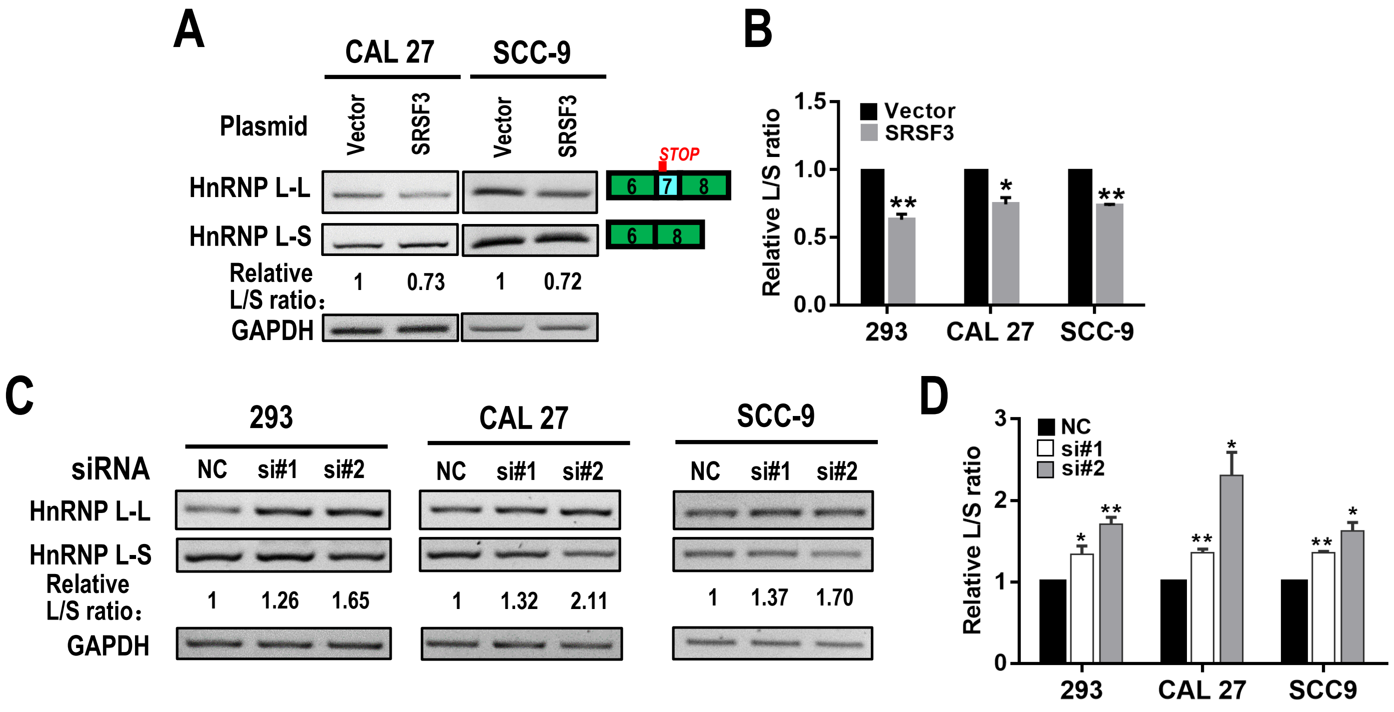

Figure S4. SRSF3 inhibits the inclusion of hnRNP L exon 7. (**A**) CAL 27 or SCC-9 cells were transfected by SRSF3 overexpression plasmid or vector control plasmid. The alternative splicing of hnRNP L exon 7 was analyzed by using two pairs of primers for isoforms with exon 7 (HnRNP L-L) or without exon 7 (hnRNP L-S), respectively. Relative L/S ratio represents the ratio of exon 7 inclusion versus exclusion isoform. (**B**) The histogram summarized the effects of SRSF3 overexpression on the alternative splicing of hnRNP L exon 7 in HEK 293, CAL 27, and SCC-9 cells. Data are the means ± SE, n = 3. (**C**) Knockdown of SRSF3 promotes the inclusion of hnRNP L exon 7 in HEK 293, CAL 27, and SCC-9 cells. Cells were transfected with anti-SRSF3 siRNA (si#1 or si#2) or non-specific control siRNA (NC). The alternative splicing of exon 7 was analyzed by RT-PCR by using two pairs of primers. *: $P<0.05$, **: $P<0.01$. (**D**) The histogram summarized the effects of SRSF3 knockdown on the alternative splicing of hnRNP L exon 7 in HEK 293, CAL 27, and SCC-9 cells. Data are the means ± SE, n = 3.

Figure S5

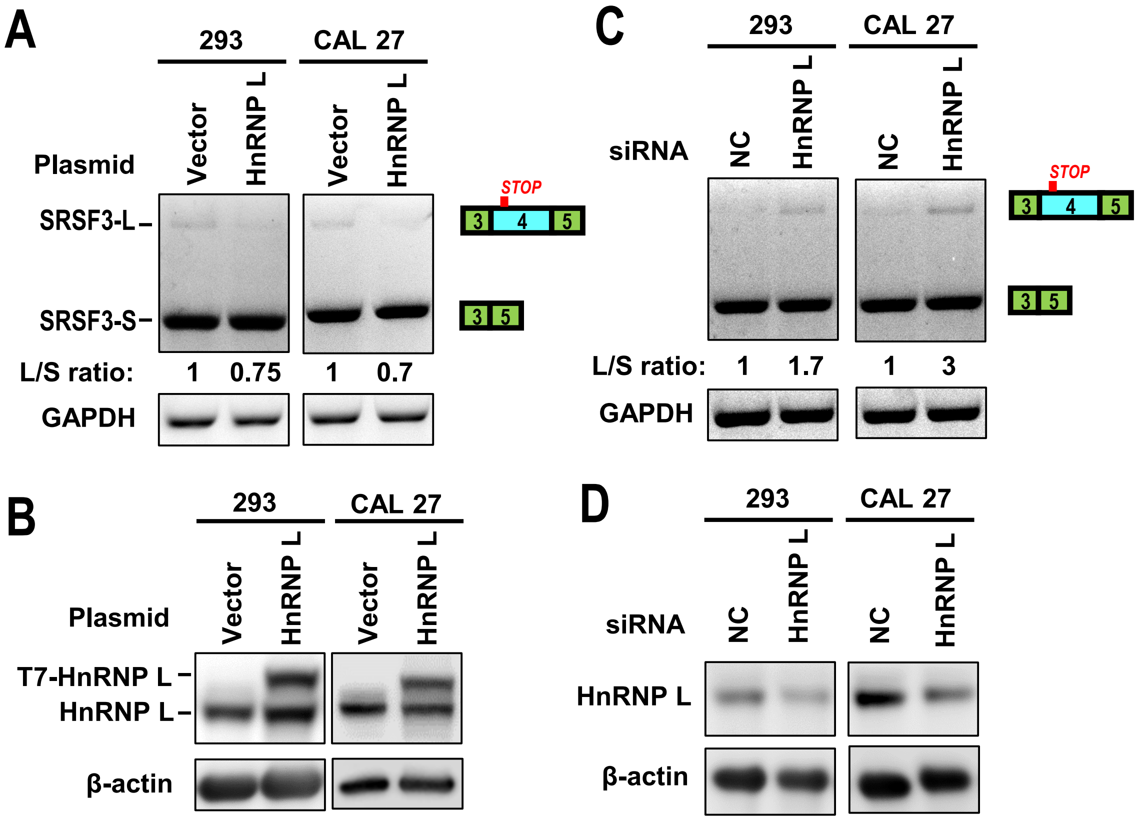

Figure S5. HnRNP L inhibits the inclusion of SRSF3 exon 4. (**A**) HEK 293 or CAL 27 cells were transfected by SRSF3 expression or vector control plasmid. The alternative splicing of exon 4 was analyzed by RT-PCR. The L/S ratio represents the ratio of exon 4 inclusion versus exclusion isoform. (**B**) The overexpression of T7-tagged hnRNP L was confirmed by Western blot. (**C**) Knockdown of hnRNP L promotes the inclusion of SRSF3 exon 4 in HEK 293 or CAL 27 cells. Cells were transfected with anti-hnRNP L siRNA or non-specific control siRNA (NC). The alternative splicing of exon 7 was analyzed by RT-PCR. (**D**) The knockdown efficiency of hnRNP L was confirmed by Western blot.

Figure S6

A

HnRNP L exon 7

5’ GGUCGCAGUGUAUGUUUGAUGGGACGCCAUCUUUCAGAACUGUGCUAACUCAC 3’

B

SRSF3 exon 4

5’
AGUCACCAUCAUGUCUCUUCUCACCACCCUCUGAAUCUGCAUUAGCCAGUCAACUAGCCCUUUCAGCGU
CAUGUGACCAGCGCGCCCCAUUCAGCUUGGCUGGUGUCGUUUCACAUGACCCAGGCUGGCCAGUCGUCA
GGUUGCACCGCCCUUUGGUUCCCGAGCAUGCUGUUUUCUCUCAGCCUUCUCUCCAACCUUAACCAAAUC
GGCAGCAGCCACCUCGACCGCCCACACAUUCCUGGCCAAUAGCUCAGCUGUUUAUUUACCAAAUGUCUU
CACAACAACUACAGCAGCAGCCUUCGGCUAACAAAAAAGCAGGAAAAAUCCACAACACCCCCUUCGCCAAC
CAACUAAAUCCAACGCAACAUCUGGCAAAACCUUUUCAGCAAAUUCUUCCUGGCCGUCAGUCCGGCAGC
CUCACCUCACCAUUUCUAGCUUGUUGAAACCCAAAACUA 3’

Figure S6. The potential binding motif of hnRNP L and SRSF3. (**A**) SRSF3 binding motif “CAUC” in hnRNP L exon 7 predicted by SpliceAid online program. (**B**) CA rich motifs in SRSF3 exon 4.

Figure S7

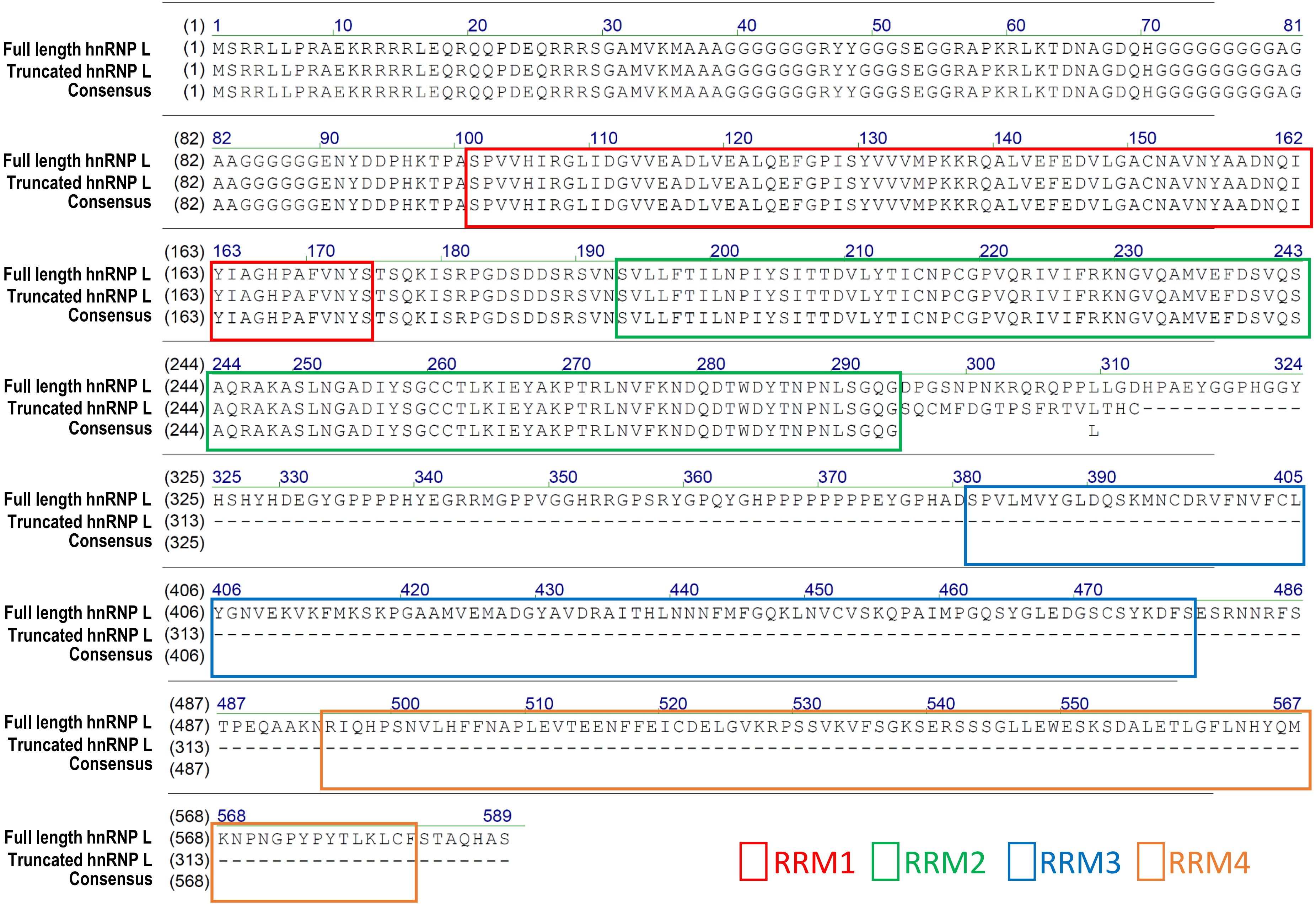

Figure S7. Alignment of full-length and truncated hnRNP L protein sequences.
