# Supplementary material for: Inclusion of hnRNP L Alternative Exon 7 Is Associated with Good Prognosis and Inhibited by Oncogene SRSF3 in Head and Neck Squamous Cell Carcinoma
Source: Biomed Res Int. 2019 Nov 13;2019:9612425. doi: 10.1155/2019/9612425 (PMC6885243; doi:10.1155/2019/9612425)
Supplement: Supplementary Materials — Figure S1: hnRNP L autoregulates its own expression by increasing the inclusion of exon 7. Figure S2: the RT-PCR results of the alternative splicing of hnRNP L exon 7 in normal or OSCC tissues. Figure S3: searching splicing factors for regulating the alternative splicing of hnRNP L exon 7. Figure S4: SRSF3 inhibits the inclusion of hnRNP L exon 7. Figure S5: hnRNP L inhibits the inclusion of SRSF4 exon 4. Figure S6: the potential binding motif of hnRNP L and SRSF3. Figure S7: alignment of full-length and truncated hnRNP L protein sequences. Table S1: clinicopathological characteristics of patient samples and L/S ratio in OSCC. [file 9612425.f1.zip › 9612425.f1/table S1.pdf]

Table S1: Clinicopathological characteristics of patient samples and L/S ratio in OSCC

**Characteristics**	**Numeber of cases（%）**
**Age(Y)**	
<55	28（45.9）
⩾55	33（54.1）
**Gender**	
Male	42（68.9）
Female	19（31.1）
**Clinical stage**	
I	4（6.6）
II	19（31.1）
III	13（21.3）
IV	25（41.0）
**T classification**	
T1	6（9.8）
T2	36（59.0）
T3	7（11.5）
T4	12（19.7）
**N classification**	
N0	31（50.8）
N1	12（19.7）
N2	18（29.5）
**Pathologic differentiation**	
high-grade	28（45.9）
low-grade	33（54.1）
**Drinking**	
NO	36（59.0）
YES	25（41.0）
**Smoking**	
NO	28（45.9）
YES	33（54.1）
**Lymphatic invasion**	
NO	33（54.1）
YES	28（45.9）
